# Damage Potential and Feeding Preference of *Halyomorpha halys* (Stål), *Nezara viridula* (L.), and *Leptoglossus zonatus* (Dallas) Among Different Ripening Stages of Tomato

**DOI:** 10.3390/insects16070740

**Published:** 2025-07-20

**Authors:** Md Tafsir Nur Nabi Rashed, Adam G. Dale, Gideon Alake, Simon S. Riley, Nicole Benda, Amanda C. Hodges

**Affiliations:** 1 Department of Entomology and Nematology, University of Florida, Room 3205, 1881 Natural Area Drive, Gainesville, FL 32611, USA; agdale@ufl.edu (A.G.D.); agideon@ufl.edu (G.A.); achodges@ufl.edu (A.C.H.); 2 Statistical Consulting Unit, Institute of Food and Agricultural Sciences, University of Florida, Gainesville, FL 32611, USA; simon.riley@ufl.edu; 3 Florida Department of Agriculture and Consumer Services, Division of Plant Industry, Gainesville, FL 32608, USA; nicole.benda@fdacs.gov

**Keywords:** brown marmorated stink bug, feeding preference, feeding puncture, feeding damage, leaf-footed bugs, southern green stink bug, ripening stages, stink bugs, tomato

## Abstract

Tomato (*Solanum lycopersicum* L.) is one of the most preferred hosts of polyphagous stink bugs (Hemiptera: Pentatomidae) and leaf-footed bugs (Hemiptera: Coreidae). These hemipterans can infest tomato fruits at all stages of fruit ripening. However, it is unclear whether there is any feeding preference for these true bugs among different ripening stages of tomato (green, breaker, pink, and red stages). Feeding and behavioral assays were performed to determine the feeding preference and damage potential of two common stink bugs—the brown marmorated stink bug (*Halyomorpha halys* (Stål)) and the southern green stink bug (*Nezara viridula* L.)—and a leaf-footed bug (*Leptoglossus zonatus* (Dallas)) among the various ripening stages of tomato. The results indicated that green is the most preferred ripening stage for *N. viridula* and *L. zonatus*, while pink tomatoes were found to be a more preferred feeding site for *H. halys*. Fully ripe red tomatoes were found to be the least preferred feeding site for all three insects. The findings of this study will be useful for developing fruit damage symptom-based monitoring programs and establishing economic threshold levels for these pests in tomatoes, as well as informing harvesting regimes.

## Simple Summary

Stink bugs and leaf-footed bugs are common insect pests of tomato that cause feeding damage by creating yellowish spots on the fruit surface. Green, unripe tomatoes undergo multiple ripening stages: the fully unripe green stage, the yellowish–green colored breaker stage, the orange-colored pink stage, and the fully ripe red stage. It has not been well studied whether stink bugs and leaf-footed bugs have a preferred tomato ripening stage to feed on. This is critical information for growers, because the most preferred ripening stage is the most vulnerable to stink bug infestation and damage. We conducted two experiments to determine the most preferred tomato ripening stage for some common stink bugs, including the Brown Marmorated Stink Bug (BMSB), the Southern Green Stink Bug (SGSB), and the Western Leaf-footed Bug (WLB). Our experiments indicated that green is the most preferred ripening stage by SGSB and WLB, while pink was found to be preferred by BMSB. Fully ripe red tomatoes were found to be the least preferred feeding site for all three insects. The findings of our study provide valuable information for developing stink bug and leaf-footed bug monitoring programs and determining when to take immediate action to manage these pests in the field.

## 1. Introduction

In 2021, the United States was listed as a leading global producer of tomatoes (*Solanum lycopersicum* L.) (Solanaceae), generating 0.97 million metric tons valued at USD 1 billion [1]. This makes tomatoes the most valued crop among all fresh vegetables cultivated in the country [2]. A wide range of arthropod pests infest tomatoes in the United States. Stink bugs (Hemiptera: Pentatomidae) are one of the common insect groups that cause economic damage to tomatoes. In Georgia, a total loss of USD 689,600 was estimated for tomatoes caused by different stink bugs in 1993 [3]. Among the 180 known species of stink bugs, six are considered pests of tomato in the United States: the southern green stink bug (*Nezara viridula* L.), the green stink bug (*Chinavia hilaris* (Say)), the brown stink bug (*Euschistus servus* (Say)), the dusky stink bug (*Euschistus tristigmus* (Say)), *Euschistus quadrator* Rolston, and the brown marmorated stink bug (*Halyomorpha halys* (Stål)) [3,4]. These stink bugs are considered pests because they often reach high densities, and prefer to feed on the reproductive parts of plants [5,6,7,8,9] by inserting needle-like stylets into plant tissue. This feeding injury causes characteristic stink bug feeding damage, appearing as “cloud spots,” “cloudy blotches,” or “blemishes.” Below the puncture, the tissues become spongy or corky due to the enzymatic reaction of stink bug saliva secreted during the feeding process [5,10]. When stink bugs feed on immature tomato fruit, the fruit matures early, reducing its size, weight, and quality [11]. In addition to direct feeding damage, stink bugs can facilitate the entry of plant pathogens through feeding wounds [11], and some have been found to transmit plant diseases mechanically [12,13]. For example, *N. viridula* was found to be responsible for causing bacterial spots and buckeye rot disease by *Phytophthora* spp. in tomatoes [11].

Several factors are known to influence the behaviors and feeding activities of stink bugs, which have implications for the severity of damage and the effectiveness of employed management tactics. Stink bugs primarily prefer to feed on the reproductive parts of plants as they are typically the most nutritious parts, but their behaviors are also influenced by various olfactory cues [9]. A study has reported that phenol, undecane, decanal, and caryophyllene are the volatiles that were associated with host plants that *H. halys* was most attracted to [9]. Although immature insects often prefer to feed on the host where they hatch, the nymphs of some stink bugs (*H. halys*, *N. viridula*, *E. servus*) have the ability to migrate among different host plants and select hosts based on volatile organic compounds associated with fruit ripening [9,14,15,16]. Therefore, it is possible that the ripening stages of the reproductive part of plants are highly related to insect infestation and subsequent feeding injury. In addition to olfactory cues, the feeding preference of stink bugs can be influenced by visual stimuli, such as the color of the potential feeding site. Several studies have focused on using visual stimuli, such as trap color, to develop trapping systems for stink bugs [17,18,19]. However, the influence of visual stimuli on the host plant or plant part selection by stink bugs is not well understood.

Tomatoes exhibit a unique flowering pattern, where their individual inflorescences develop sequentially, extending their bloom period and resulting in clusters of tomato fruits containing different ripening stages at the same time [5]. Based on the color of tomato fruits in different ripening stages, the United Fresh Fruit and Vegetable Association, in collaboration with the US Department of Agriculture and the Agricultural Marketing Service Fruit and Vegetable Division, has developed a set of standards for assessing the grade of tomatoes [20]. This grading system classifies fresh tomatoes into six distinct categories: green stage (fruit surface is fully green), breaker stage (a definite break in color, which should not be more than 10% of the fruit surface), turning stage (fruit surface is 10–30% tannish–yellow, pink, red, or a combination of these colors), pink stage (30–60% of the fruit surface shows a pink or red color in the aggregate), light red stage (60–90% of the fruit surface is pinkish–red or red-colored), and red stage (more than 90% of the fruit surface is red) [20]. The variation in tomato color is a result of a combination of various pigments accumulated in the epidermis, the sub-epidermal layer, and the pericarp of the tomato, and is regulated by a wide range of biochemical reactions [21]. However, color is not the sole differentiating factor among the different ripening stages of tomatoes. Fruits at each ripening stage emit different levels and types of volatiles [22,23]. The volatiles associated with tomato fruit flavor increase with the ripening time from the breaker stage to full ripeness [22,24]. Additionally, the volatile concentration also increases with ripeness, making the odor stronger [25,26]. A study isolated 181 volatiles from the mature green stage, 221 from the breaker stage, 240 from the pink stage, and 367 from the red stage of tomato [25]. Moreover, around 400–412 aroma compounds have been identified as the major compounds responsible for the aroma and flavor of fresh ripe tomatoes [27,28]. These volatiles and visual cues likely influence Hemiptera colonization, feeding behaviors, and subsequent pest pressure in tomato production systems [9].

Feeding preferences among various ripening stages have been observed previously in some other crops. *Nezara viridua* and rice stink bug (*Oebaus pugnax* F.) (Hemiptera: Pentatomidae) were found to prefer to feed on the milk stage of wheat kernels than the soft-dough stage [29], and preferred full-sized soybean pods (R6 stage) over small-sized pods (R3 stage) [30]. Moreover, *E. quadrator* Rolston adults have been reported to prefer to feed on green blackberries (*Rubus* spp.) than on ripe blackberries [19]. Another study found that *H. halys* preferred the orange-colored bell pepper over green-, yellow-, and red-colored bell peppers of the same variety, representing the different ripening stages of bell peppers [31].

The influence of the tomato ripening stage on stink bug damage potential is not well studied. A field study reported that *Euschistus conspersus* Uhler (Hemiptera: Pentatomidae) preferred to feed on green tomatoes over red ones; however, no data were presented to support this information [32]. Moreover, another field study in 1960 reported that *N. viridula* preferred to feed on red tomato fruits over green ones, but did not provide any experimental evidence [5]. On the other hand, a 2012 study reported that *N. viridula* nymphs exhibited a preference for green tomatoes, while adults mostly targeted red ones; however, this was also a field observation without any experimental evidence [33]. A lab investigation reported that both male and female *N. viridula* preferred green tomato fruits over red ones of the same size, and spent a longer time in proboscis insertion on green tomato fruit than on red fruit [5]. However, they did not include the other ripening stages in the laboratory experiment. A recent study reported that light red and red tomato fruits contained significantly more stink bug feeding punctures than green, breaker, turning, and pink stages of tomato in a sorghum trap cropping field experiment [4]. However, as the fruits were picked at the same time, red or light red tomatoes were in the field with stink bugs for a longer period than those in other stages of ripening. Thus, it cannot be concluded from this study that red tomatoes are more vulnerable to damage by stink bugs. To fill this knowledge gap, a feeding preference bioassay and a behavioral assay were conducted. We set out to address the following objectives:

1. Determine the feeding preference of different piercing–sucking hemipteran pests (stink bugs and leaf-footed bug) among four major ripening stages (green, breaker, pink, and red) of tomato fruit, in order to find out which ripening stage is most preferred by stink bugs.
2. Determine whether the feeding preferences of piercing–sucking hemipteran pests for the tomato ripening stages vary among different developmental stages (nymphs, adult males, and adult females).
3. Investigate the damage potential among different piercing–sucking hemipteran pests in tomato.

For this study, two invasive stink bug species were used—the southern green stink bug *N*. *viridula* and the brown marmorated stink bug *H*. *halys*. Although *N. viridula* is a cosmopolitan polyphagous pest, tomato is one of its primary and most economically affected hosts. Tomato is also the most preferred host for *H. halys,* compared to other vegetable crops [10]. This study also investigated the feeding preference of a leaf-footed bug species, *Leptoglossus zonatus* (Dallas) (Hemiptera: Coreidae). *Leptoglossus zonatus* causes similar feeding damage to tomato fruits as the stink bug pests. This species is highly polyphagous and is currently recognized as an emerging pest within the Gulf Coast region of the United States, posing a severe threat to Satsuma mandarin (*Citrus unshiu*) (Rutaceae) [34,35,36]. However, a study has reported that *L. zonatus* prefers to feed on tomatoes over the other regional crops, including peaches, lemons, and Satsuma mandarins [36].

## 2. Materials and Methods

### 2.1. Insect Collection and Rearing

Colonies of *N. viridula* and *L. zonatus* were established in 2021 at the Biosecurity Research and Extension Laboratory at the University of Florida’s Entomology and Nematology Department (UF-ENY) in Gainesville, Florida, USA. Founding individuals were collected from multiple locations in Alachua and Marion Counties in Florida. Colonies of *H. halys* were established in 2023 at the Florida Biological Control Laboratory Quarantine Facility at the UF-ENY, which were sourced from the Phillip Alampi Beneficial Insect Rearing Laboratory, New Jersey Department of Agriculture, Trenton, NJ, USA. *Nezara viridula* and *L. zonatus* were reared in butterfly habitat cages (39.88 cm × 39.88 cm × 60.96 cm; BioQuip, Compton, CA, USA). *Halyomorpha halys* was reared in a smaller cage (30.48 cm× 30.48 cm × 30.48 cm; Restcloud) placed inside a larger cage (39.88 cm × 39.88 cm × 60.96 cm; BioQuip) for biosecurity purposes. All insects were reared on organic sugar snap peas (*Pisum sativum* L.) (Fabaceae), baby-cut carrots (*Daucus carota* L.) (Apiaceae) (Funayama, 2006), raw peanuts (*Arachis hypogaea* L.) (Fabaceae), and corn cobs (*Zea mays* L.) (Poaceae). Food materials were replenished with fresh food three times a week. The colonies were maintained at 26 ± 2 °C, 60 ± 5% relative humidity, and a photoperiod of 16L:8D [37]. Each cage was scouted for egg masses three times a week. Collected egg masses were placed on a filter paper (90 mm diameter, medium porosity, Fisherbrand^TM^ Quantitative Grade Filter Paper Circles, Thermo Fisher Scientific Inc., Waltham, MA, USA) in a small Petri dish (100 mm × 15 mm, Fisherbrand^TM^ Petri Dishes with Clear Lid, Thermo Fisher Scientific Inc., Waltham, MA, USA) with a sugar snap pea and placed in a separate butterfly habitat cage. The nymphs were maintained in that cage for the remainder of their development, following the protocol described earlier. When the nymphs reached the fourth instar, the number of nymphs per cage was counted and 100 nymphs per cage were maintained. If there were more than 100 nymphs, extra nymphs were transferred into a new cage with a camel hairbrush. Each cage was cleaned every two weeks by removing all the debris from the cage.

### 2.2. Growing Greenhouse Tomatoes

For this study, 144 indeterminate grape tomatoes (Johnny’s, FIVE STAR GRAPE F1 OG, Certified Organic by MOFGA, Johnny’s Selected Seeds, Winslow, ME, USA) were planted through the transplanting technique in a greenhouse at the UF-ENY. Seeds were planted in multiple seed trays (9 cm × 9 cm × 5 cm per cell) in the summer of 2023. Three weeks later, the seedlings were transplanted into plastic pots (22.86 cm × 22.86 cm) using Miracle-Gro Potting Mix (N:P:K = 0.21:0.11:0.16). When the plants were about 45 cm tall, a 180 cm-long bamboo stake was placed in each pot and tied to the plant for support, which is essential for obtaining high-quality fruit, increasing yield, and making the plant easier to manage [38,39]. Three weeks after transplanting, each plant was pruned by removing two lower-bottom suckers, which increases tomato yield per plant and reduces the risk of plant disease dispersal [40]. Leaf pruning was also performed as necessary, in order to increase air and light circulation in the bush and reduce the risk of disease incidence [41]. The plants were scouted weekly for disease and insect pests. No disease or insect pests were observed during the study period.

### 2.3. Experimental Arenas (Figure 1)

For the feeding and behavioral assays, a circular transparent plastic arena (15.24 cm diameter and 6.35 cm deep) with a lid was used. The arena was modified by cutting four circular 3.80 cm-diameter openings in the sides and covering them with fine mesh using glue to ensure air circulation inside the arena. The lid of the arena was also ventilated by cutting a circular 3.80 cm-diameter opening and covering it with fine mesh fabric. A circular paper towel (15.24 cm in diameter) was placed at the bottom of the arena. The arena was divided into four quadrants by folding the circular paper towel, which made four-fold marks on the paper towel. Each quadrant had an equal area with a 90° angle at the center. One experimental insect was randomly caught from the colony and was gently released at the center of an arena. For both assays, one insect was used per arena. For the collection and handling, a camel hair paintbrush and a plastic container were used to avoid mechanically injuring or stressing the insect. Then, the insect was kept in the arena for 12 h before the tomato fruits were introduced, in order to acclimate and starve them before starting the assays. The same temperature (26 ± 2 °C), relative humidity (60 ± 5%), and photoperiod (16L:8D) were maintained in both assays, as employed for the stink bug colonies in this study.

### 2.4. Experiment 1: Feeding Preference Bioassay

After 12 h of starvation in the arenas, four freshly harvested tomatoes—each representing a different ripening stage (green, breaker, pink, and red)—were gently placed into the arena, one per quadrant. The tomatoes used for each replicate were harvested from a single tomato plant. Tomato ripening stages were categorized using the USDA tomato grading standards color reference chart [20] and verified through visual assessment. Prior to introduction, each fruit was evaluated to ensure there were no existing feeding punctures or signs of other insect damage or disease. The tomatoes selected for each arena were approximately the same size, although fruit size has been shown not to influence stink bug feeding [5]. A precise timetable was maintained for this experiment: the 12-h insect resting period was from 8 pm to 8 am, which included 4 h of photophase time and 4 h of scotophase. The tomatoes were introduced in the arena at 9 am, and the insects were allowed to feed on the tomatoes for 24 h. Since stink bug feeding activity can differ between photophase and scotophase [10,42,43], each bug was allowed to feed on tomatoes for one full photophase and scotophase. After 24 h, the tomatoes were removed from the arenas, and the number of feeding punctures on each fruit was counted under a stereomicroscope. A dot was applied to every puncture using a Sharpie^®^ pen (Shelbyville, TN, USA), ensuring that none of the feeding punctures were counted twice. This experiment was replicated 18 times for each life stage (adult female, adult male, and 4th instar nymph) of each insect species (*N. viridula*, *H. halys*, and *L. zonatus*). Each arena was considered a replicate.

### 2.5. Experiment 2: Behavioral Assay

The behavioral assay followed a similar protocol as the feeding assay, but focused on recording the feeding behavior of the insects. After 12 h of starvation, one tomato of each of four different ripening stages (green, breaker, pink, and red) was carefully placed into the four quadrants of the arena. Then, insects were allowed to choose among the four tomatoes for one hour. One hour later, the ripening stage of the tomato that the insect was feeding on in the arena was recorded at one-hour intervals for the next 12 h. This 12-h period included the photophase only. This experiment was replicated 15 times for each life stage (adult female, adult male, and 4th instar nymph) of each species (*N. viridula*, *H. halys*, and *L. zonatus*). Each arena was considered a replicate containing only one insect.

### 2.6. Data Analysis

The damage potential of stink bugs, measured by the number of punctures, was analyzed separately for each stink bug species using a generalized linear mixed model (PROC GLIMMIX) in SAS OnDemand for Academics (Version 15.1, Copyright 2023, SAS Institute, Cary, NC, USA). The number of punctures was modeled as a function of the ripening stage, using a negative binomial distribution. The least squares mean number of feeding punctures was calculated for each ripening stage and for each stink bug species, and pairwise comparisons between ripening stages were made using Tukey’s adjustment for multiple comparisons (α = 0.05). Confidence limits (95%) were calculated for all least squares means. Data from the behavioral assay were analyzed using R version 4.4.0 [44]. The proportion of time that experimental insects spent feeding at each ripening stage was modeled as being binomially distributed (with a logit link function) by fitting a generalized linear mixed model [45] using the glmmTMB package [46]. Species, ripening stages, and sex, along with all higher-order interactions, were included as random effects, while the individual insect was included as a random effect. Model fit was assessed via a visual inspection of the quantile residuals [47]. Model fitting was followed by an F-test, calculation of least square means, and separation of means using the emmeans package [48].

## 3. Results

### 3.1. Feeding Bioassay

**Figure 2:**
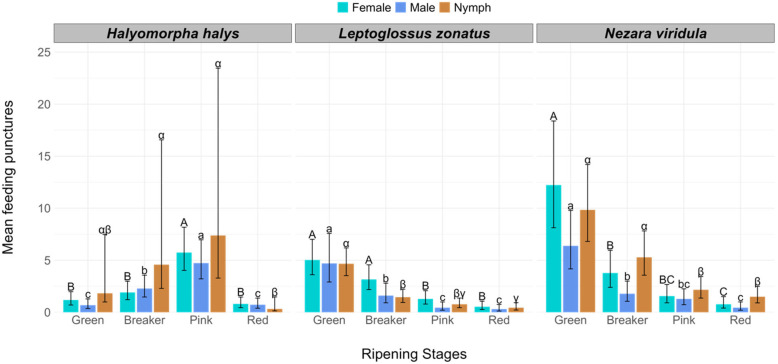
Mean number of feeding punctures caused by females, males, and nymphs of *Halyomorpha halys*, *Nezara viridula*, and *Leptoglossus zonatus* on tomato fruit at different ripening stages. Comparisons are made among the same life stages within each species, where females are compared with majuscule English letters, males with minuscule English letters, and nymphs with minuscule Greek letters.

This bioassay was conducted to determine which stage of tomato ripening was most preferred by the experimental insects. Male and female *H. halys* caused more feeding punctures on pink tomatoes than on those at any other ripening stage (green, breaker, and red) (F = 25.05; *p* ≤ 0.0001) (Table 1, Figure 2). *Halyomorpha halys* nymphs produced a mean number of 7.38 (5.30) punctures on the pink tomatoes, which was higher than that of adult life stages. However, no difference was detected in nymph feeding punctures among green, breaker, and pink tomatoes (F = 25.05; *p* ≤ 0.0001). All three life stages of *H. halys* produced the fewest feeding punctures on red and green tomatoes (Table 1, Figure 2). On the other hand, *N. viridula* females, males, and nymphs caused more feeding punctures on green tomatoes than on pink and red tomatoes (Table 1, Figure 2) (F = 50.51; *p* ≤ 0.0001). On average, *N. viridula* nymphs deposited a mean number of 9.83 (0.84) salivary sheaths per green tomato; however, this was not significantly different than the number of salivary sheaths deposited per breaker tomato (Figure 2). Similarly to *N. viridula*, *L. zonatus* females, males, and nymphs caused the most damage to green tomatoes and the least to the red tomatoes (Table 1, Figure 2). However, no differences were found between the number of feeding punctures on green tomatoes and breaker tomatoes caused by female *L. zonatus* and, for all three life stages, no difference was found in the number of feeding punctures in pink and red tomatoes (Figure 2).

**Figure 3:**
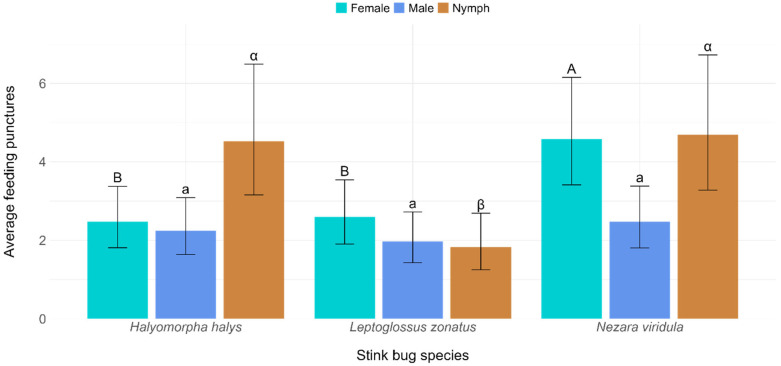
Mean number of feeding punctures caused by *Halyomorpha halys*, *Nezara viridula*, and *Leptoglossus zonatus* on tomato fruits. Comparisons are made among the same life stages, where females are compared with majuscule English letters, males with minuscule English letters, and nymphs with minuscule Greek letters.

Among the three heteropteran species, nymphs of *H. halys* and *N. viridula* caused significantly more damage than *L. zonatus* (F = 7.81; *p* = 0.0005) (Table 2, Figure 3). However, there was no significant difference between the mean number of feeding punctures caused by the two pentatomid species. On the other hand, female *N. viridula* produced more feeding punctures than female *H. halys* and *L. zonatus,* but no significant difference was observed between the number of feeding punctures caused by female *H. halys* and *L. zonatus* (Figure 3). No significant difference was observed among the males of the three species, in terms of the number of feeding punctures (F = 0.49; *p* = 0.6108).

### 3.2. Behavioral Assay

**Figure 4:**
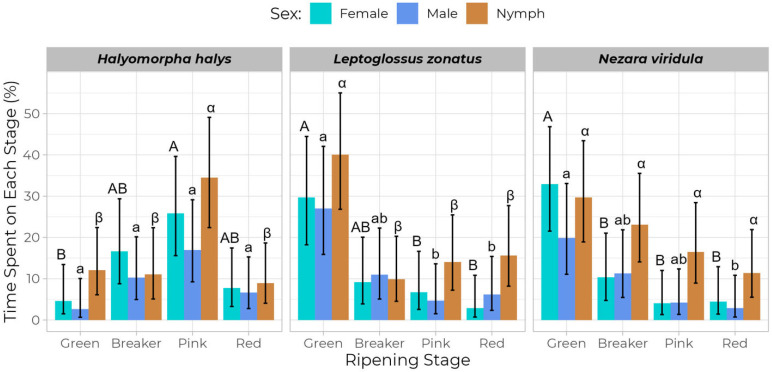
The average percentage of feeding time of *Halyomorpha halys*, *Nezara viridula*, and *Leptoglossus zonatus* on tomato fruit at different ripening stages. Comparisons are made among the same life stages within each species, where females are compared with majuscule English letters, males with minuscule English letters, and nymphs with minuscule Greek letters.

The behavioral assay revealed that life stage and sex have an influence on the time spent by *H. halys* on different ripening stages (F = 12.87; *p* = 2.56 × 10^−6^). Nymphs of *H. halys* spent more time on pink tomatoes than on the other ripening stages during the total 12 h of feeding (F = 8.22; *p* = 1.81 × 10^5^). Female *H. halys* spent more time on pink tomatoes than on green ones, while males spent a moderate amount of time on pink fruits but overall spent less time on green ones (Figure 4). On the other hand, female *N. viridula* were found to spend more time (32.96 ± 6.57%) on the green tomatoes, but no difference was observed for breaker, pink, and red tomatoes (Figure 4). *Nezara viridula* males exhibited a consistent behavior of spending time on different ripening stages and showed no significant difference between breaker, pink, and red stages (F = 0.82; *p* = 0.632). Similarly, *N. viridula* nymphs spent more time on green tomatoes (29.73 ± 6.35%), but this was not significantly different than the time spent on the other ripening stages (Figure 4). *Leptoglossus zonatus* nymphs spent significantly more time (40.11 ± 7.38%) on green tomatoes than breaker, pink, and red tomatoes (F = 8.22; *p* = 1.81 × 10^−5^). Female *L. zonatus* also spent more time on green tomatoes, but the difference was not significant with respect to the breaker stage, and no difference was observed for breaker, pink, and red tomatoes (Figure 4). Similarly, male *L. zonatus* spent more time on green tomatoes, but no difference was observed for breaker, pink, and red ripening stages (F = 0.99; *p* = 0.427).

## 4. Discussion

Our study revealed that the feeding activities of different stink bugs and leaf-footed bugs vary depending on the ripening stages of tomato fruits. *Halyomorpha halys* showed a strong preference for the pink ripening stage over fully ripe red and unripe green tomatoes. During the ripening process, the accumulation of β-carotene creates an orange color during the pink ripening stage of tomato fruit [49]. It has been found that β-carotene is an attractive visual cue for various insects [50,51]. Moreover, it was previously reported that *H. halys* preferred orange bell peppers—which have a similar color to pink tomatoes—over the other ripening stages of bell peppers (green, yellow, and red) [31]. Therefore, the preference for the pink tomatoes by *H. halys* could be related to the β-carotene content or the orange color during the pink ripening stage.

On the other hand, our study recorded a similar dominant preference for green tomatoes over red ones in *N. viridula*, as reported by Lye and Story [5]. Moreover, an interesting trend of *N. viridula* and *L. zonatus* in terms of feeding preference was observed, where the number of feeding punctures was found to decrease with the ripeness of the tomato fruits (Figure 2). This preference by *N. viridula* and *L. zonatus* for less ripe fruit could be due to the different types and concentrations of plant volatile compounds that increase with tomato ripeness. The volatile compounds associated with increasing tomato ripeness may include some defensive volatiles that repel *N. viridula* and *L. zonatus*, resulting in fewer feeding punctures on red tomatoes compared to green, unripe tomatoes. However, further research is needed to determine whether there is a specific group of volatile compounds that repel or alter the feeding behaviors of *N. viridula* and *L. zonatus* on red tomatoes. This could be very useful information for the development of different lure traps to manage these pests by utilizing those potential volatile compounds.

This study also determined that *N. viridula* has a greater damage potential than *H. halys* and *L. zonatus,* as *N. viridula* produced more feeding punctures on tomatoes. The number of average feeding punctures was found to be similar to those reported in some previous studies [5,52]. Moreover, our study also confirmed that the life stage and sex of the stink bugs and the leaf-footed bug have an influence on feeding activity, as they produced different levels of feeding punctures and spent varying amounts of feeding time across different ripening stages. For example, in the behavioral assay, the nymphs of all three species exhibited a stronger and clearer preference for a particular tomato ripening stage, while males generally showed a more generalized preference. However, the feeding performance of stink bugs at different life stages can vary depending on the species and developmental stage of the host plant [30,53,54]. In our study, the nymphs of *N. viridula* and *H. halys* caused more feeding punctures on tomatoes than the adults. This might happen because nymphal stink bugs prefer to feed on a particular site for a long time without moving much due to their aggregation behavior, while adult stink bugs are highly mobile and can change their feeding site more frequently [55,56]. This was also supported by our behavioral experiment, where we observed that nymphs spent more time at a particular ripening stage than adults (Table 3). *Leptoglossus zonatus* was found to be less destructive to tomatoes than the other species, as it produced fewer feeding punctures (Table 2, Figure 3). This finding aligns with a previous study reporting that *H. halys* caused more damage than *L. zonatus* [57]. However, the extent of damage caused by a stink bug species can vary depending on the host species [10].

## 5. Conclusions

Tomato plants set fruit for an extended period. Therefore, all the ripening stages (green, breaker, turning, pink, light red, and red) can exist at the same time in the field. For this reason, it is crucial for growers to know which ripening stage is most prone to stink bug damage. Knowledge on which ripening stage is most susceptible to damage can be used to determine the damage threshold at different stink bug density levels. Our findings offer valuable insights for enhancing monitoring systems and establishing an economic threshold level of stink bugs through fruit scouting. Compared to other crops, monitoring for stink bugs in tomatoes is quite challenging due to the insects’ high mobility [3]. Sweep nets and beat sampling are effective methods for monitoring stink bugs [58,59]. However, these tools cause damage to plants, affecting the quality and yield of tomato fruits. Utilizing pheromone lure traps for stink bug monitoring is an effective technique; however, due to the associated cost and maintenance requirements, it is impractical for growers to implement this method throughout every growing season. In this study, we found that *N. viridula* and *L. zonatus* caused more damage to green tomatoes than those in other stages, while *H. halys* preferred pink tomatoes. Therefore, it would be wise and time-efficient for growers to focus on green and pink tomatoes, rather than randomly scouting tomato fruit at any ripening stage, in order to detect the presence of common stink bug pests in the field. Further research is needed to determine the efficacy of this scouting method and establish an action threshold level. Moreover, tomato plants with fruits at specific ripening stages, such as green or pink, may be deployed as effective sentinel plants, acting as early detection tools for monitoring stink bug activity in the field. Furthermore, this study provides valuable insights for the development of trap cropping systems, specifically through adjusting the planting dates of trap crops. By implementing these findings, growers can enhance the effectiveness of their tomato crop management strategies against stink bugs. Additionally, the findings of this research offer initial insights towards the development an attract-and-kill trapping system to manage *H. halys*, *N. viridula,* and *L. zonatus* by mimicking the desired ripening stage through color and volatiles; for example, using an insecticide-treated artificial surface or a 3D-printed tomato-shaped sphere to be implemented with an attract-and-kill methodology, as previously successfully used to manage other insect pests [60,61]. However, this study was conducted under controlled laboratory conditions using a single tomato variety in order to minimize variability. Hence, future studies should evaluate whether feeding preferences vary across different tomato cultivars and under diverse environmental conditions to enhance the applicability of this study’s results for field-level management.

## Figures and Tables

**Figure 1:**
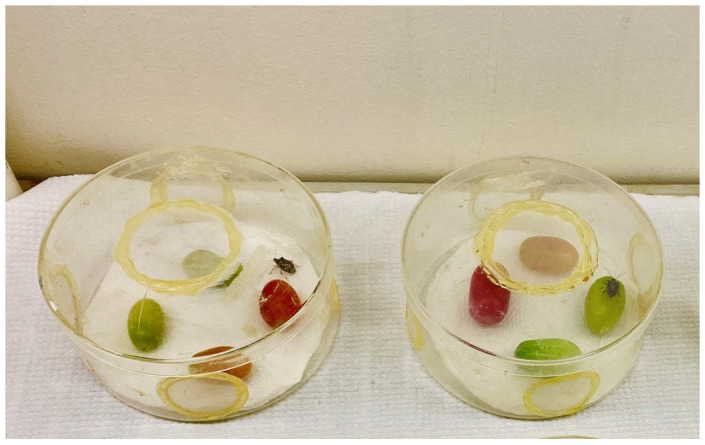
Experimental arena setup for feeding and behavioral assays.

**Table 1:** Mean (±SE) number of feeding punctures caused by females, males, and nymphs of *Halyomorpha halys*, *Nezara viridula*, and *Leptoglossus zonatus* on tomato fruit at different ripening stages (green, breaker, pink, and red).

Tomato Ripening Stage	Mean (±SE) Feeding Punctures by *Halyomorpha halys*			Mean (±SE) Feeding Punctures by *Nezara viridula*			Mean (±SE) Feeding Punctures by *Leptoglossus zonatus*		
	Female	Male	Nymph	Female	Male	Nymph	Female	Male	Nymph
Green	1.18 ± 0.31	0.68 ± 0.22	1.83 ± 1.40	12.22 ± 2.50	6.39 ± 1.36	9.83 ± 1.81	5.03 ± 0.84	4.70 ± 1.12	4.67 ± 0.66
Breaker	1.90 ± 0.43	2.28 ± 0.51	4.57 ± 3.34	3.78 ± 0.86	1.78 ± 0.46	5.28 ± 1.04	3.15 ± 0.58	1.59 ± 0.45	1.44 ± 0.31
Pink	5.73 ± 1.02	4.73 ± 0.92	7.38 ± 5.30	1.56 ± 0.42	1.28 ± 0.36	2.17 ± 0.50	1.28 ± 0.31	0.45 ± 0.18	0.78 ± 0.22
Red	0.81 ± 0.24	0.73 ± 0.23	0.32 ± 0.28	0.78 ± 0.26	0.44 ± 0.18	1.50 ± 0.38	0.54 ± 0.18	0.29 ± 0.14	0.44 ± 0.16

**Table 2:** Mean (±SE) total number of feeding punctures caused by *Halyomorpha halys*, *Nezara viridula*, and *Leptoglossus zonatus* on tomato fruits.

Sex	*Halyomorpha halys*	*Nezara viridula*	*Leptoglossus zonatus*
Female	2.47 ± 0.39	4.58 ± 0.69	2.60 ± 0.41
Male	2.25 ± 0.36	2.47 ± 0.39	1.97 ± 0.32
Nymph	4.52 ± 0.83	4.69 ± 0.86	1.83 ± 0.36

**Table 3:** Mean (±SE) percentage of feeding activity time of *Halyomorpha halys*, *Nezara viridula,* and *Leptoglossus zonatus* on tomato fruit at different ripening stages (green, breaker, pink, and red).

Tomato Ripening Stage	Mean (±SE) Percentage (%) of Feeding Activity Time of *Halyomorpha halys*			Mean (±SE) Percentage (%) of Feeding Activity Time of *Nezara viridula*			Mean (±SE) Percentage (%) of Feeding Activity Time of *Leptoglossus zonatus*		
	Female	Male	Nymph	Female	Male	Nymph	Female	Male	Nymph
Green	4.63 ± 2.62	2.64 ± 1.86	12.05 ± 4.03	32.96 ± 6.57	19.88 ± 5.60	29.73 ± 6.35	29.69 ± 6.81	27.02 ± 6.78	40.11 ± 7.38
Breaker	16.67 ± 5.18	10.29 ± 3.72	11.05 ± 4.22	10.32 ± 3.96	11.28 ± 4.02	23.10 ± 5.50	9.16 ± 3.87	11.01 ± 4.20	9.90 ± 3.82
Pink	25.83 ± 6.20	16.99 ± 5.02	34.53 ± 6.96	4.07 ± 2.32	4.22 ± 2.40	16.49 ± 4.91	6.75 ± 3.26	4.69 ± 2.65	14.02 ± 4.55
Red	7.80 ± 3.36	6.69 ± 2.94	8.97 ± 3.52	4.43 ± 2.51	2.87 ± 2.01	11.34 ± 4.03	2.87 ± 2.01	6.19 ± 3.01	15.61 ± 4.90

## Data Availability

The article includes the original contributions presented in this study. Further inquiries can be directed to the corresponding author.
